# Supplementary material for: SETBP1 induces transcription of a network of development genes by acting as an epigenetic hub
Source: Nat Commun. 2018 Jun 6;9:2192. doi: 10.1038/s41467-018-04462-8 (PMC5989213; doi:10.1038/s41467-018-04462-8)
Supplement: Supplementary file 1 — Supplementary Information [file 41467_2018_4462_MOESM1_ESM.pdf]

SETBP1 induces transcription of a network of development genes by acting as an epigenetic hub

Piazza et al.

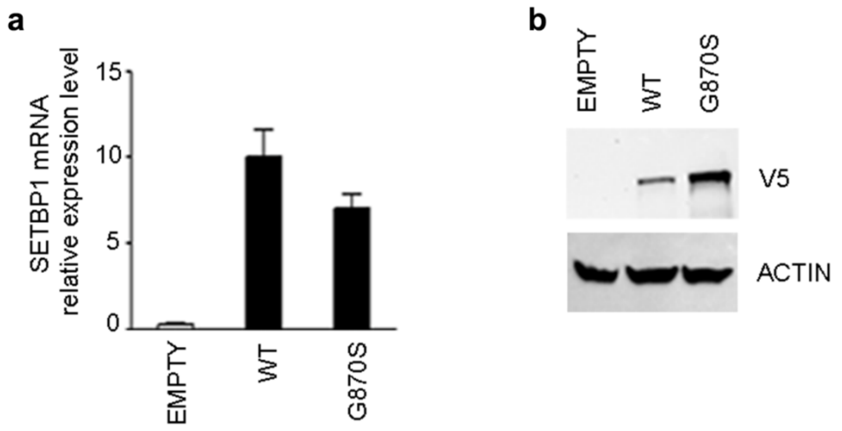

Supplementary Figure 1: Characterization of 293 FLP-In stable transfectants. a) Q-PCR on 293 FLP-In cells expressing WT or mutated (G870S) SETBP1. The housekeeping gene *GUSB* was used as an internal reference. b) Western Blot analysis of 293 FLP-In total cell lysates. V5 antibody recognizes the SETBP1-V5 tagged form expressed from the pFRT-SETBP1 vector. Actin was used as loading control.

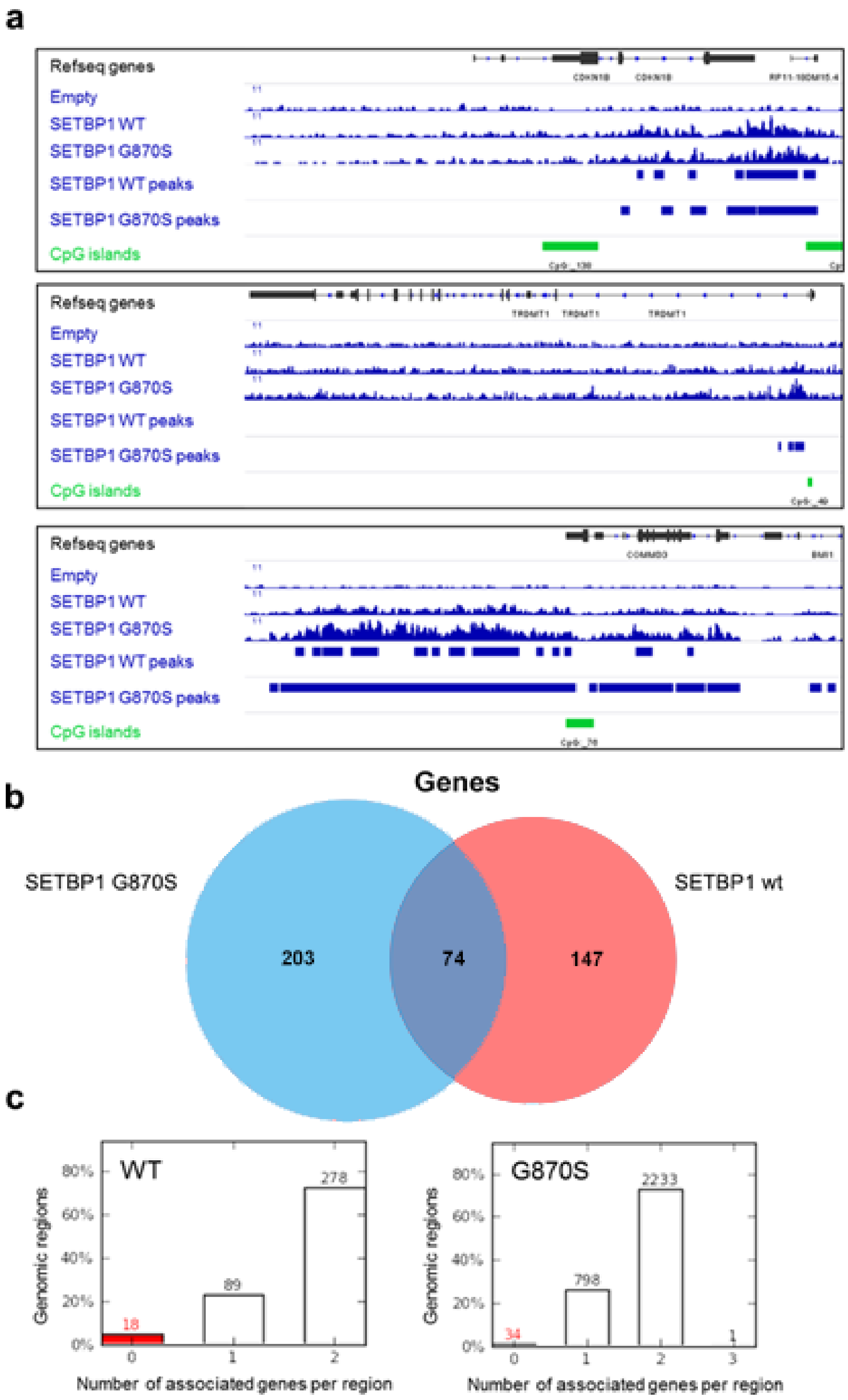

Supplementary Figure 2: Genomic regions bound by SETBP1. a) Examples of SETBP1 ChIP-Seq coverage tracks and peak alignments to the hg19 reference genome. CpG islands are highlighted in light green. b) Venn diagram displaying the intersection between genes bound by WT and G870S SETBP1. c) Number of associated genes per single region bound by WT and G870S SETBP1 highlighting the high specificity of the performed binomial peak calling.

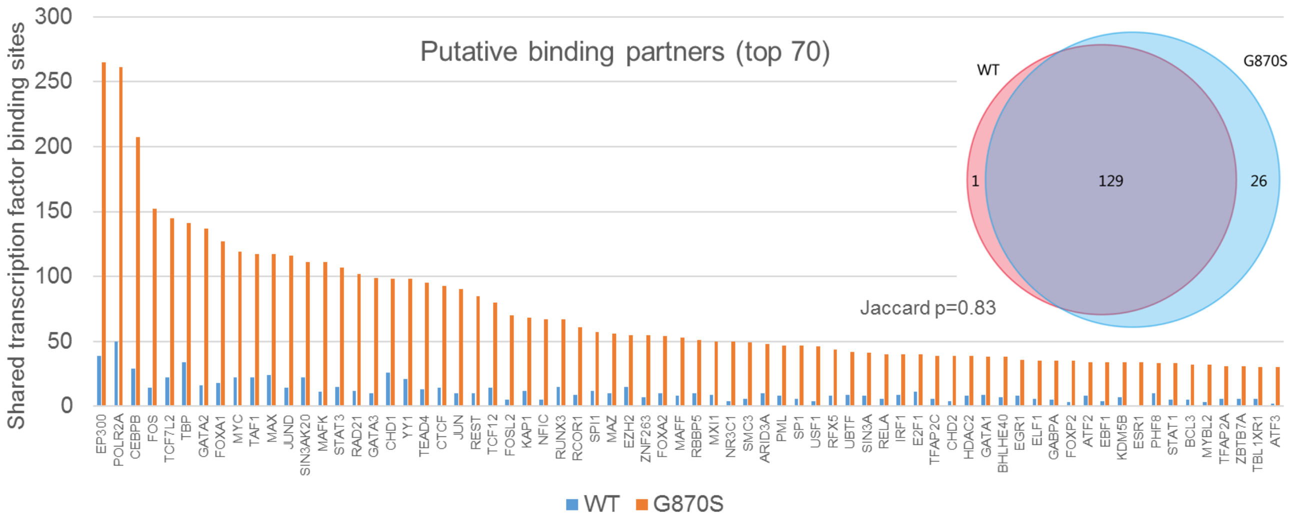

Supplementary Figure 3: Intersection between SETBP1 ChIP-Seq peaks for SETBP1-WT and G870S and ENCODE transcription factor binding sites (TFBSs). The histogram represents the number of shared transcription factor binding sites between SETBP1-WT and G870S; the Venn diagram represents the intersection between genes whose promoter is occupied by SETBP1-WT (red) and those occupied by SETBP1-G870S (cyan).

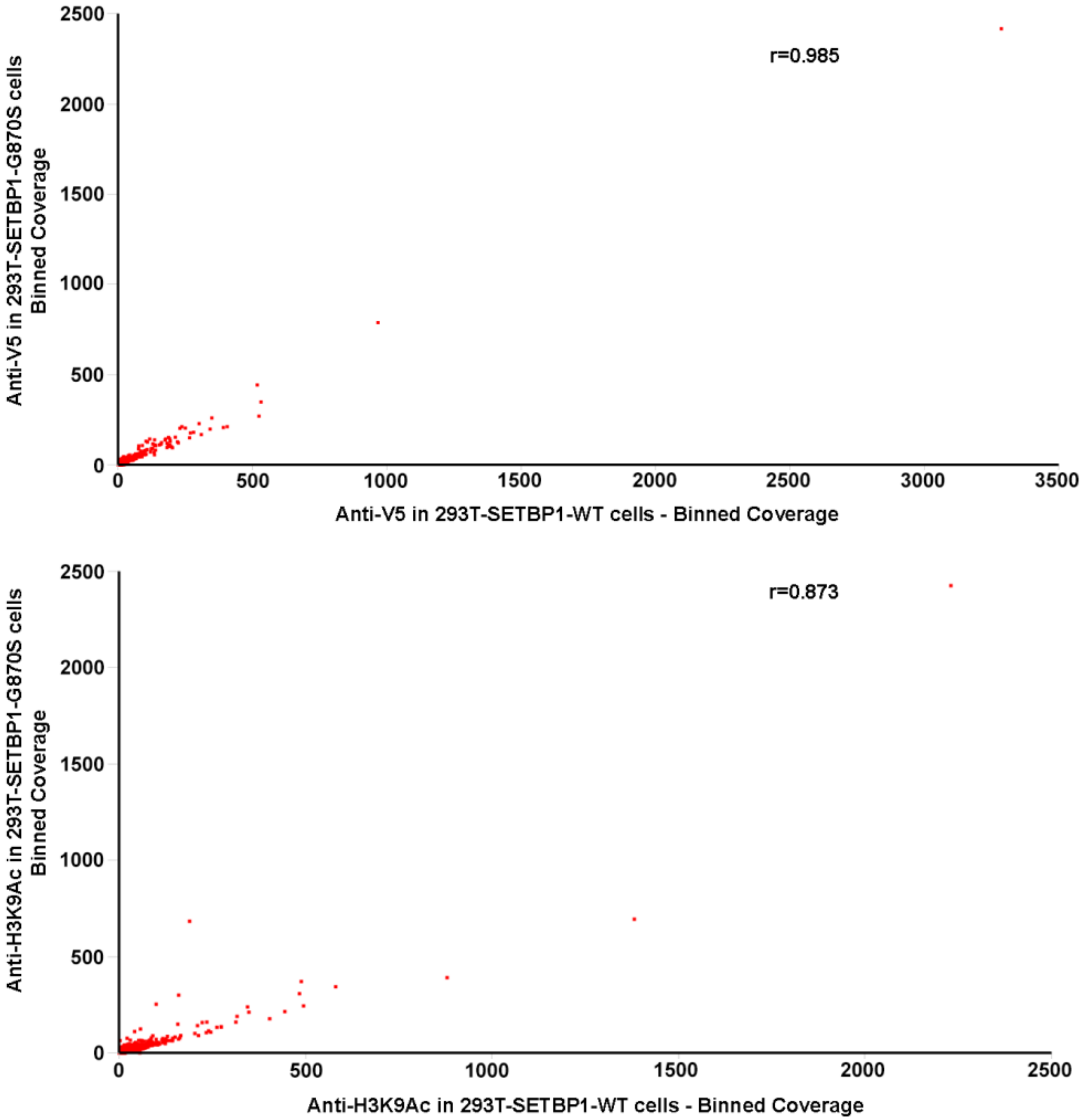

Supplementary Figure 4: Plot showing the linear correlation between the binned coverage of ChIP-Seq experiments directed against the V5 tag (upper panel) or the H3K9Ac epigenetic mark in SETBP1-WT (x axis) and G870S cells (y axis). r = Pearson correlation coefficient.

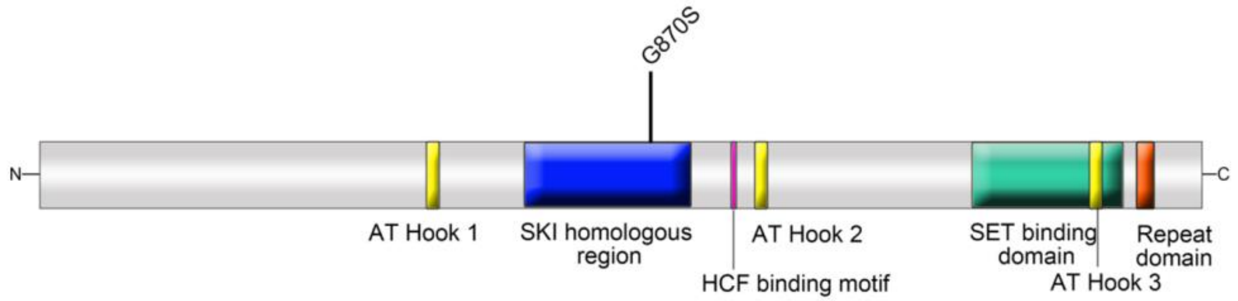

Supplementary Figure 5: Schematic representation of SETBP1 protein. The predicted HCF1 binding motif (HBM) (aa991-994) is indicated in pink. The AT Hooks are indicated in yellow.

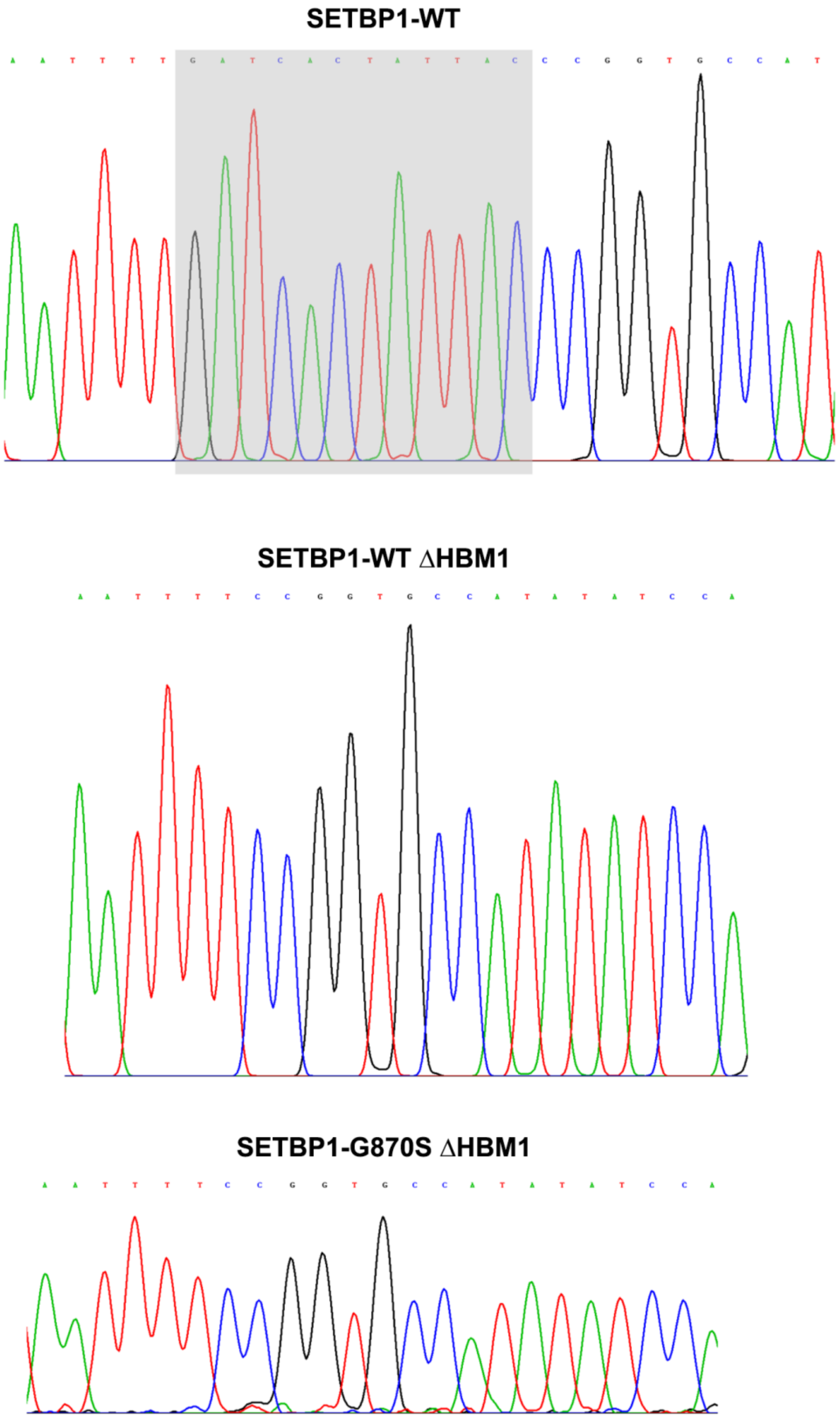

Supplementary Figure 6: **Sanger sequencing of pcDNA6.2 SETBP1.** WT (upper panel), WT ΔHBM (central) and G870S ΔHBM (lower panel) are shown. The shadowed area indicates the deleted nucleotides, corresponding to the HBM (aa 991-994).

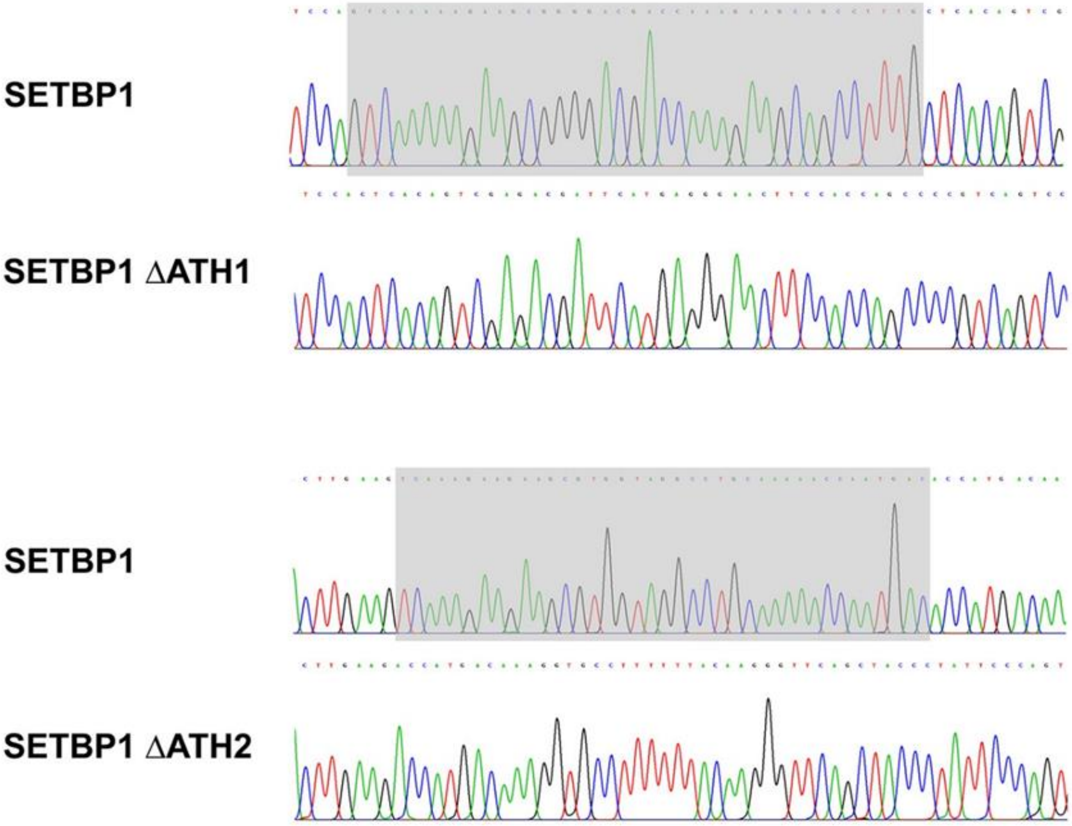

Supplementary Figure 7: **Sanger sequencing of pEF5/FRT/SETBP1.** Results of Sanger sequencing performed on SETBP1 ΔATH1 and ΔATH2 are shown. The shadowed areas in the reference sequence indicate the deleted nucleotides, corresponding to the ATH1 (aa 584-596) or ATH2 (aa 1016-1028).

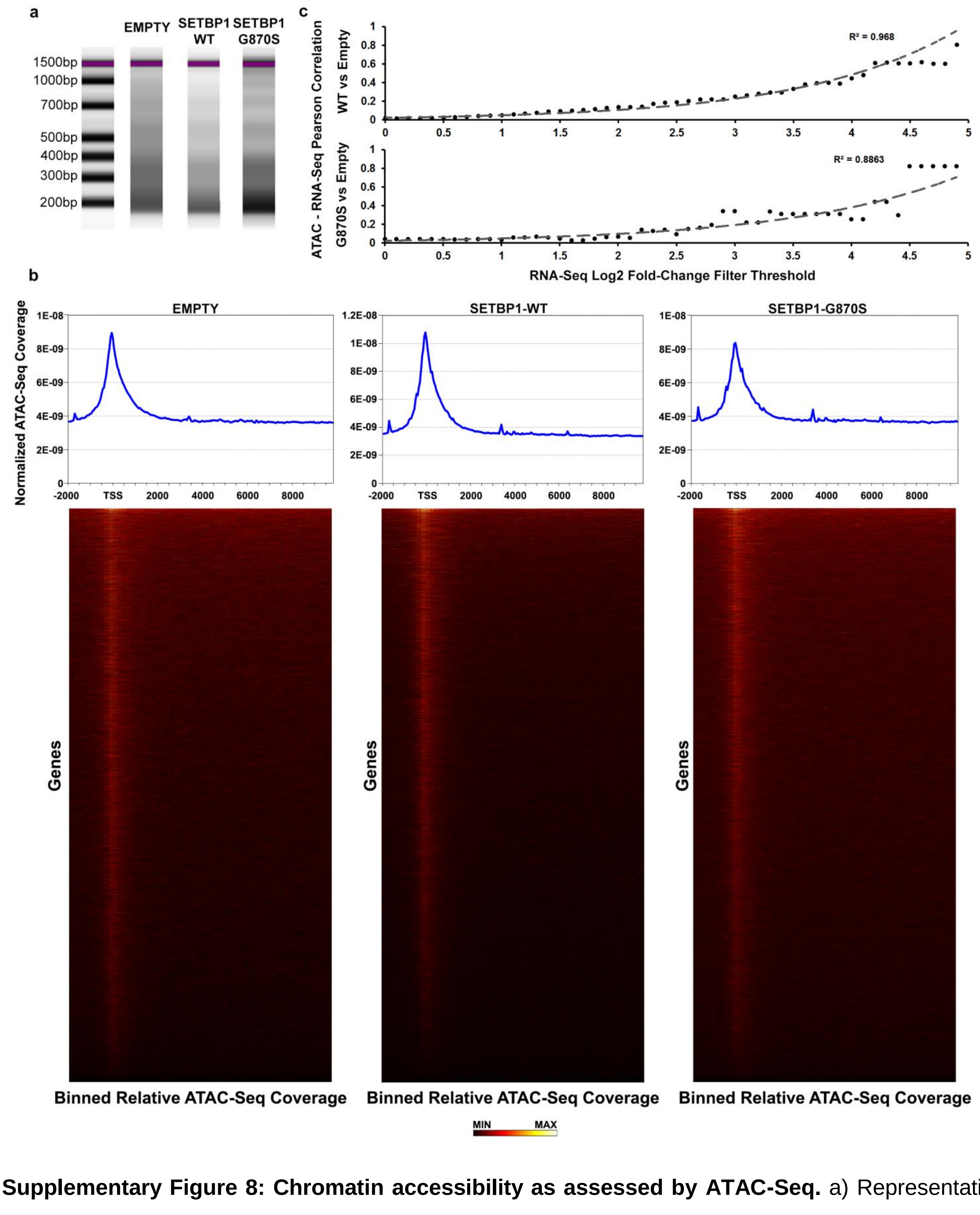

Supplementary Figure 8: Chromatin accessibility as assessed by ATAC-Seq. a) Representative image of three ATAC-Seq libraries showing the expected banding pattern. The first lane represents the gel marker. b) The upper plot shows the normalized, cumulative ATAC-Seq signal for 293 FLP-In Empty, SETBP1-WT and SETBP1-G870S in a region comprised between -2000 and +10000 from the

TSS of all the human genes. The lower plot (heatmap) shows the normalized, binned ATAC-Seq signal for the same cell lines. Each line of the heatmap represents a gene. Genes are ordered according to the global (from -2000bp to +10000bp) intensity of the ATAC-Seq signal. c) Pearson correlation (R score; vertical axis) between the ATAC-Seq relative, normalized signal (SETBP1-G870S vs Empty and SETBP1-WT vs Empty) and the RNA-Seq relative gene expression in presence of increasing Log2 Fold-Change RNA-Seq filter values (SETBP1-G870S vs Empty and SETBP1-WT vs Empty; horizontal axis). Each dot represents the result of a single Pearson correlation at a given Log2 Fold-Change Filter (0 to 4.9).

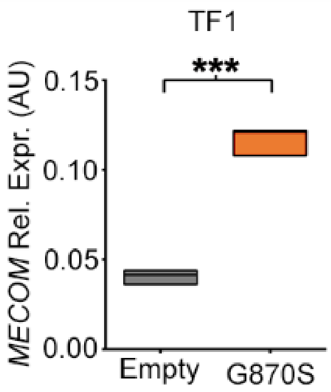

Supplementary Figure 9: Q-PCR analysis of *MECOM* expression in the TF1 cell line transduced with an empty vector or with a vector encoding SETBP1-G870S. The top and bottom of each box represent the first and third quartile; the internal line represents the median.

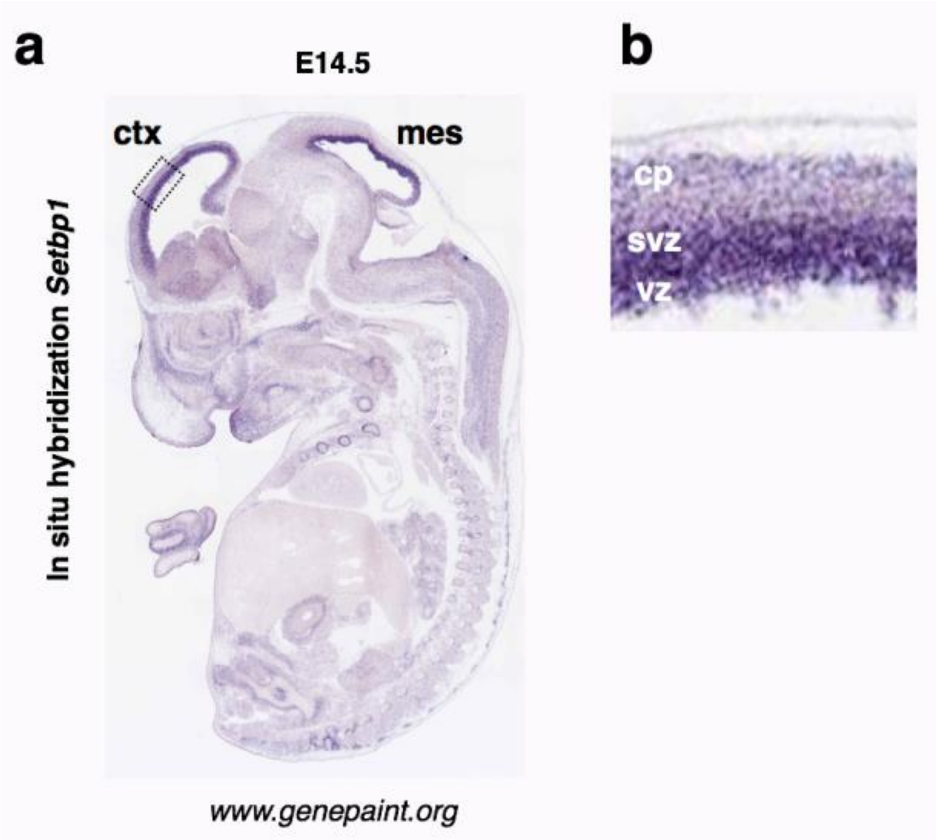

Supplementary Figure 10: Setbp1 expression in mouse embryo. a) *In situ* hybridization revealed the Setbp1 mRNA as particularly present in developing nervous system of E14.5 mouse embryo. Ctx = cortex, mes = mesencephalon. b) Setbp1 is abundant in germinal layers of cortex as evident from magnification. cp= cortical plate, iz = inner zone, svz = subventricular zone, vz = ventricular zone. The images are from www.genepaint.org. GenePaint set ID: MH396.

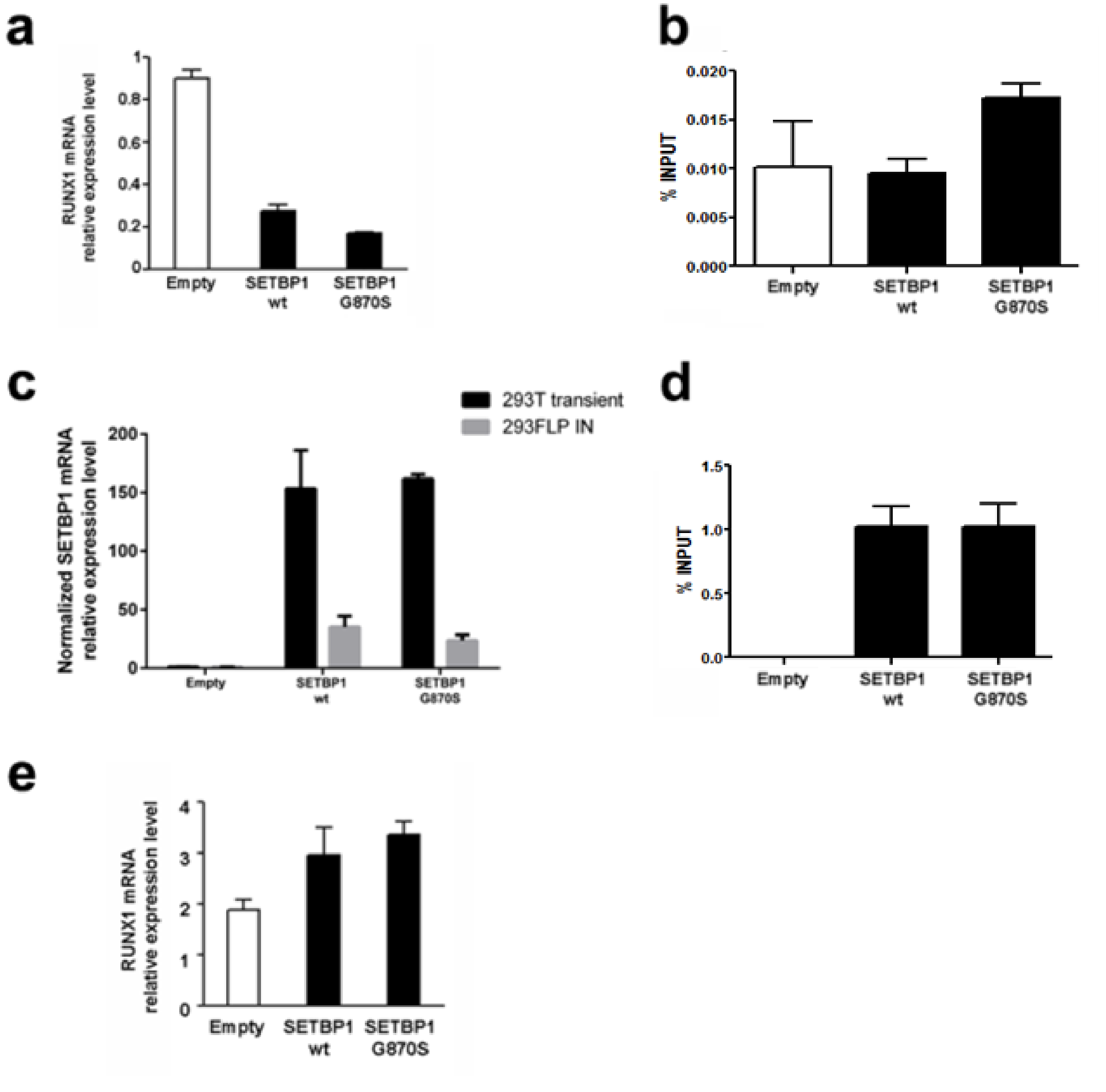

Supplementary Figure 11: Activity of SETBP1 on RUNX1 promoter. a) *RUNX1* mRNA expression level in stable transfectants (293 FLP-In); b) ChIP showing SETBP1 binding on *RUNX1* proximal promoter (P2) in stable transfectants analyzed through Q-PCR. Values are normalized on INPUT. c) *SETBP1* expression level in transient (293T) and stable (293 FLP-In) transfectants. Values are normalized on the corresponding Empty cells. d) ChIP results for SETBP1 binding on *RUNX1* proximal

promoter (P2) in transient transfectants analyzed through Q-PCR; values are normalized on INPUT. e) *RUNX1* mRNA expression level in transient transfectants (293T).

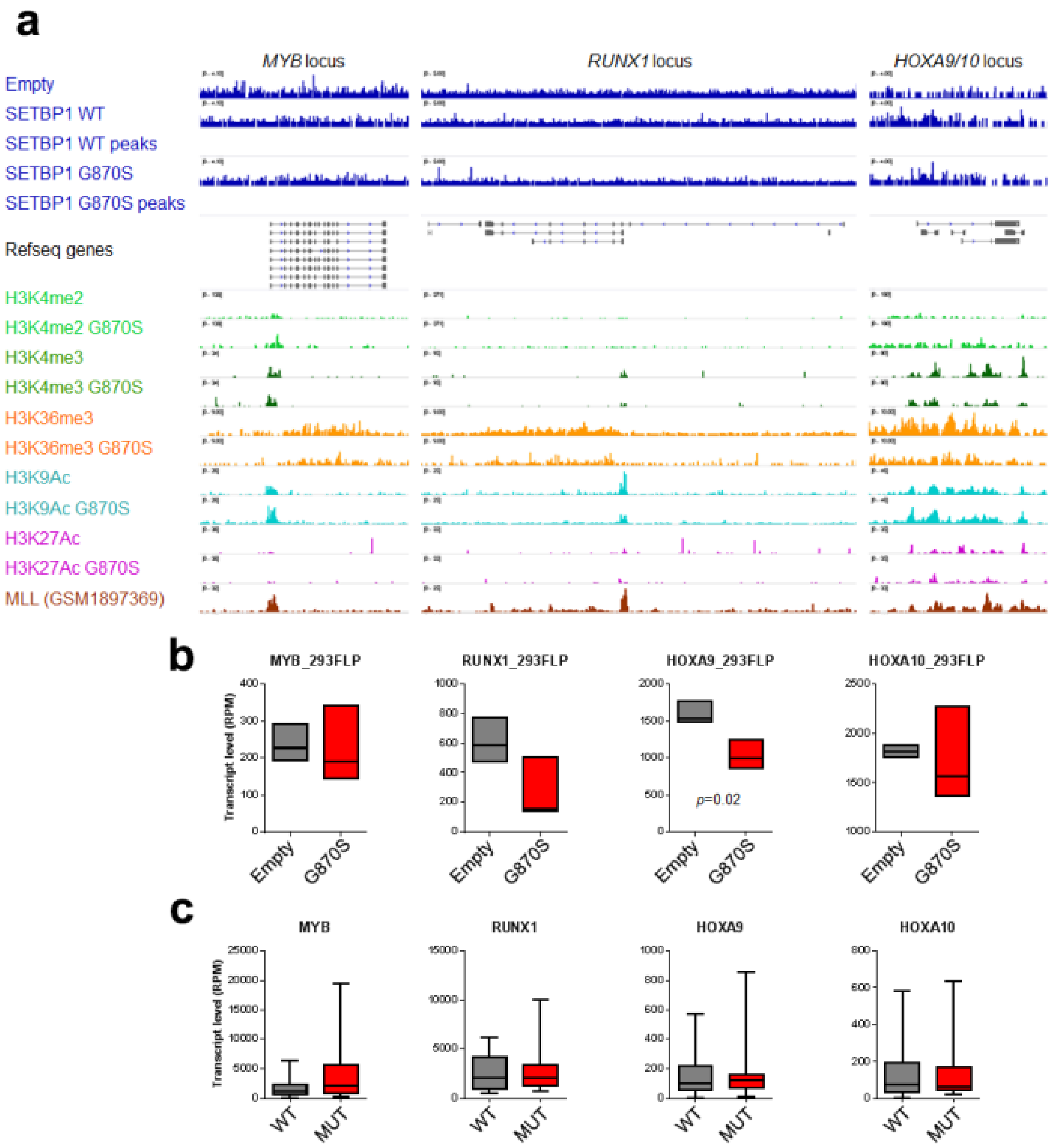

Supplementary Figure 12: Analysis of *MYB*, *RUNX1* and *HOXA9/10* loci. a) ChIP-Seq coverage tracks and peak alignments to the hg19 reference genome for *MYB*, *RUNX1* and *HOXA9/10* loci. b) Relative expression of *MYB*, *RUNX1*, *HOXA9* and *HOXA10* in Empty and SETBP1-G870S FLP-In lines. c) Relative expression of *MYB*, *RUNX1*, *HOXA9* and *HOXA10* in 32 aCML patients, 11 positive and 21 negative for SETBP1 somatic mutations.

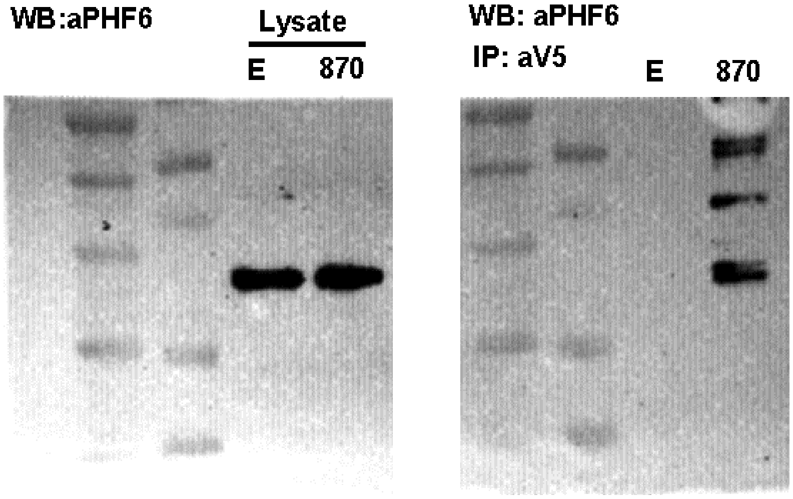

Supplementary Figure 13: uncropped scan of the western blots related to PHF6 immunoprecipitation.
