# Supplementary material for: SETBP1 induces transcription of a network of development genes by acting as an epigenetic hub
Source: Nat Commun. 2018 Jun 6;9:2192. doi: 10.1038/s41467-018-04462-8 (PMC5989213; doi:10.1038/s41467-018-04462-8)
Supplement: Supplementary file 3 — Description of Additional Supplementary Files [file 41467_2018_4462_MOESM3_ESM.pdf]

Description of Additional Supplementary Files

File Name: Supplementary Movie 1
Description: (SM1_GFP): 3D reconstruction resulting from 2-photon microscopy on GFP electroporated cortices (2 days) after tissue clarification (X-Clarity system).

File Name: Supplementary Movie 2
Description: (SM2_G870S): 3D reconstruction resulting from 2-photon microscopy on SETBP1 electroporated cortices (2 days) after tissue clarification (X-Clarity system).

File Name: Supplementary Data 1
Description: Genomic regions bound by SETBP1-G870S.

File Name: Supplementary Data 2
Description: Genes whose promoter is ectopically bound by SETBP1-G870S.

File Name: Supplementary Data 3
Description: List of 2687 genes differentially expressed between SETBP1-G870S and Empty 293 FLP-In lines.

File Name: Supplementary Data 4
Description: Genes differentially expressed in SETBP1-G870S vs. Empty 293 FLP-In lines whose promoter is occupied by SETBP1-G870S.

File Name: Supplementary Data 5
Description: Differentially expressed genes characterized by increase in H3K4me2 and H3K9ac marks.

File Name: Supplementary Data 6
Description: Co-immunoprecipitation/mass-spectrometry data.

File Name: Supplementary Data 7
Description: Binned (200bp), normalized ATAC-Seq data for 293 FLP-In Empty.

File Name: Supplementary Data 8
Description: Binned (200bp), normalized ATAC-Seq data for 293 FLP-In SETBP1-WT.

File Name: Supplementary Data 9
Description: Binned (200bp), normalized ATAC-Seq data for 293 FLP-In SETBP1-G870S.

File Name: Supplementary Data 10
Description: MECOM target genes involved in hematopoietic stem-cell proliferation and myeloid differentiation.
